# The combinatorial complexity of cancer precision medicine

**DOI:** 10.18632/oncoscience.66

**Published:** 2014-07-23

**Authors:** Frederick Klauschen, Michael Andreeff, Ulrich Keilholz, Manfred Dietel, Albrecht Stenzinger

**Affiliations:** ^1^ Institute of Pathology, Charité University Hospital Berlin, Berlin, Germany; ^2^ Department of Leukemia, Division of Cancer Medicine, The University of Texas MD Anderson Cancer Center, Houston, TX, USA; ^3^ Charité Comprehensive Cancer Center, Charité University Hospital Berlin, Berlin, Germany; ^4^ Institute of Pathology, University of Heidelberg Medical School, Heidelberg

**Keywords:** Precision Medicine, Combination Therapies, Systems Medicine, Personalized Therapy, Clinical Trial Design

## Abstract

Precision medicine approaches have recently been developed that offer therapies targeting mainly single genetic alterations in malignant tumors. However, next generation sequencing studies have shown that tumors normally harbor multiple genetic alterations, which could explain the so far limited successes of personalized medicine, despite considerable benefits in certain cases. Combination therapies may contribute to a solution, but will pose a major challenge for clinical trials evaluating those therapies. As we discuss here, reasons include the low abundance of most of the relevant mutations and particularly the combinatorial complexity of possible combination therapies. Our report provides a systematic and quantitative account of the implications of combinatorial complexity for cancer precision medicine and clinical trial design. We also present an outlook on how systems biological approaches may be harnessed to contribute to a solution of the complexity challenge by predicting optimal combination therapies for individual patients and how clinical trial design may be adapted by combining and extending basket and umbrella design features.

## INTRODUCTION

The standard approach to evaluating the utility of novel therapies in clinical studies is subject to an elaborate regulatory framework[1, 2]. Traditionally, novel therapies, for instance chemotherapy regimens, are designed based on expert knowledge and basic biological knowledge of the mode of action of the respective drugs, e. g. of DNA replication and cell proliferation. In personalized medicine, however, more and more therapies target specific, pathologically altered cellular signaling components. Compared to conventional chemotherapy the appropriate use of these novel approaches requires more in-depth knowledge of cellular mechanisms. Because most of such alterations are present only in a subset of tumors, patients' tumors need to be tested prior to therapy selection[3]. A number of these so-called “companion diagnostics” have already been established for certain cancers including chronic myeloid leukemia, melanoma, breast, gastric, colorectal and non-small-cell lung cancer. While in case of CML the presence of the fusion gene BCR-ABL is pathognomonic of the disease[4] and in case of melanoma about 60% of the tumors contain the relevant BRAF V600E mutation[5, 6], activating EGFR mutations and EML4-ALK gene fusions are present in slightly over 10% and less than 5%, respectively, of non-small cell lung cancer patients[7]. This low incidence poses a challenge to testing targeted drugs in clinical trials, because of the difficulties recruiting sufficient numbers of patients. This is illustrated by the clinical trial providing initial evidence for the utility of Crizotinib in EML4-ALK positive lung cancer comprising 105 study-centers in 27 countries but with only 347 patients[8] or another study that found only 44 ALK-, 14 RET- and 13 ROS1-fusions in 1,529 lung adenocarcinomas[9]. Moreover, despite survival benefits, these single-drug targeted therapies have not yet led to a cure in most cases calling for more sophisticated approaches targeting different pathways simultaneously. In principle, international collaboration between study centers, concepts such as decentralized patient recruiting, a modified N-of-1 clinical trial design[10] or so-called “basket trials” recruiting patients with certain common molecular properties across tumour entities[11],[12] may allow this “low-incidence” personalized medicine to be evaluated in trials of single-drug regimens[13]. Such trials will likely become increasingly relevant in the future, because it has recently been shown by us and others that mutational profiles exist across conventional cancer entities[14-16]. In an alternative approach, “umbrella trials” recruit patients with a specific cancer but different actionable mutations. The first BATTLE (Biomarker-integrated Approaches of Targeted Therapy for Lung Cancer Elimination) trial, for instance, has evaluated 4 different targeted therapies in lung cancer patients whose tumors harbor the corresponding actionable mutations[17, 18]. A second umbrella-phase 2 trial, BATTLE-2[19], has recently been launched to investigate predictive biomarkers for double drug combinations targeting EGFR, MEK and PI3K/AKT in refractory NSCLC. A slightly different adaptive phase 2 trial design was implemented for the I-SPY 2 TRIAL[20, 21] (Investigation of Serial Studies to Predict Your Therapeutic Response with Imaging And moLecular Analysis) which investigates the efficacy of targeted drugs added to conventional neo-adjuvant chemotherapy in women with locally advanced breast cancer. In this trial selection of investigational drugs is determined by biomarker screenings. While these novel phase 2 trial designs are a great leap forward in implementing cancer precision medicine, severe problems will develop even with these approaches if alternative combination therapies are to be evaluated because of the arising combinatorial complexity in conjunction with the often low frequencies at which the relevant actionable mutations occur.

In this perspective we present a quantitative analysis of the combinatorial complexity of personalized medicine and its implications for future clinical trial design. We propose a paradigm shift in the design of clinical trials from a primarily statistical to a more mechanistic approach calling for a focus on systems medicine to address disease complexity. As detailed below this major change will become necessary to reconcile the evaluation of complex targeted combination therapies designed for individual patients with the clinical trial design requirements demanded by regulatory authorities.

Low frequencies of actionable pathologic alterations, such as, activating mutations in kinases or their involvement in gene fusions, as exemplified above, are not the exception but rather the rule, as collaborative next generation sequencing efforts such as the International Cancer Genome Consortium (ICGC) have shown recently. While different cancer entities exhibit certain common patterns, they demonstrate not only strong inter-entity, but also significant intra-entity variations of their mutational profiles[14, 22, 23]. Bioinformatic and experimental studies suggest that only a fraction of the observed mutations are causally linked with cancer (driver mutations) and probably the majority of mutations can be considered bystanders without functional relevance (reviewed in [24]). However, with on average 360 exon mutations observed in the recently published data on lung cancer by The Cancer Genome Atlas (TCGA)[25], the relevance of rarer mutations (the “long tail” of infrequently mutated genes) is still not understood and even if only the most frequently mutated genes (the “head”) may be classified as drivers[26], their precise contributions to the pathologically altered cellular network function are largely elusive. While certain more frequent mutations are likely to be good target candidates there is currently no evidence ruling out less frequent mutations as contributors to disease progression and relapse in individual patients. On the contrary, given the often diverse responses to targeted therapies of tumors harboring the same mutation, it is rather likely that the patterns of less frequent, variable mutations contribute to the limited efficacy of many targeted mono-therapies. But how can one – given the diverse genetic aberrations – decide which therapy combination to choose and how to systematically evaluate such therapies in clinical trials? As we will demonstrate, this is practically impossible within conventional study designs.

The complexity rendering conventional clinical trial designs inappropriate for evaluating such combination therapies has two aspects: the combinatorial complexity of drug combinations and the low abundance of many actionable mutations. Let us consider a scenario in the not too distant future, in which 10 independent, but not mutually exclusive mutations exist that are known to be relevant for lung cancer progression. Moreover, compounds targeting each gene products are available. In a standard clinical trial approach experts would choose one or two drug triplets out of the 10 to be tested against each other and/or conventional therapy. A sufficient number of patients whose tumors harbor the mutations targeted by the triple-combination therapies would then have to be recruited. And this is where the two complexity issues potentiate each other: first, 120 different alternative triple-combination therapies exist for 10 actionable mutations (as given by the binomial coefficient $\left(\begin{array}{c} 10\\ 3 \end{array}\right)$. Second, if we consider a frequency of 20% for each of the mutations, the likelihood of a patient showing the respective three mutations is 0.8%, i. e. 25,000 patients would have to be screened (their tumor DNA would have to be sequenced) to find 200 that would fit into the study. A systematic test of all 120 possible drug combinations to find the optimal triple-therapy for 10 actionable mutations with only 200 patients per study arm would require 24,000 patients to be treated in a clinical trial.

**Figure 1:**
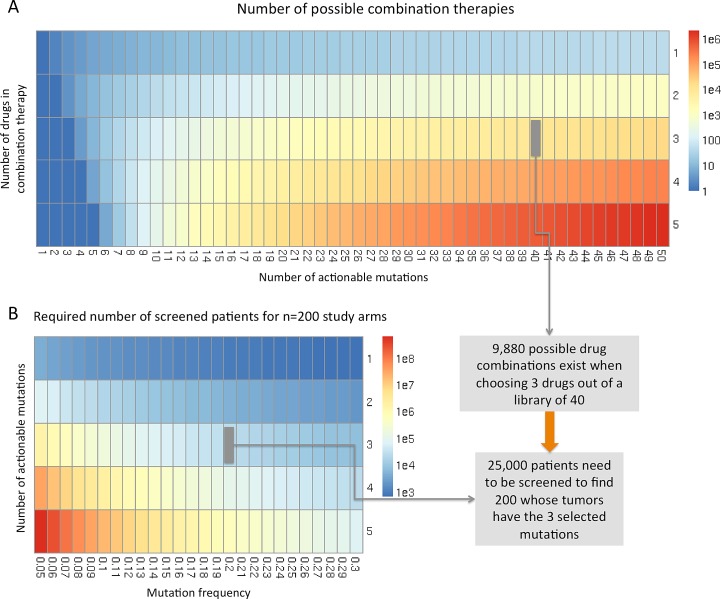
Combinatorial complexity of combination therapies in personalized medicine A: Heatmap visualization of the number of possible combination therapies in dependence on the number of actionable mutations and number of combined drugs. As an example a combination therapy with 3 drugs selected out of a set of 40 compounds yields 9,880 possible combination therapies that would have to be evaluated clinically. B: Heatmap visualization of the number of patients that would have to be screened (i. e. whose tumors would have to be sequenced) to recruit 200 patients into a clinical trial evaluating a combination therapy in dependence on the number of actionable mutations targeted in the combination therapy and the frequency at which they occur in tumors.

While it would be possible to reduce these large numbers by excluding a number of combinations based on existing knowledge of pathway redundancy, the above parameters can still be considered rather conservative given the whole genome sequencing data now available on tumors. With the above mentioned 360 exon mutations found in an TCGA lung cancer study and with potentially 10% driver mutations and a constantly growing number of available compounds for targeted therapy (for example, there are currently over 50 compounds in the pipelines of Novartis and Pfizer alone), and frequencies below 20% for EGFR- and EML4ALK-mutations the above scenario is likely to underestimate the complexity clinical trial designs will have to deal with. A still reasonable scenario of a combination therapy consisting of only five drugs chosen for a set of 50 actionable mutations yields 2,118,760 possible combinations (Figure 1A). 625,000 thousand tumors would need to be sequenced to recruit 200 patients for a 5-drug-combination targeting a 20% mutation frequency, Figure 1B). Readers skeptic of the practical relevance of such astronomical numbers might argue that it will likely be possible to exclude a significant number of drug combinations based on knowledge of pathways or pre-clinical studies using cell culture or animal experiments[27]. While such complexity reduction may certainly be possible to some extent, the dilemma is that the complexity of testing such approaches pre-clinically is increasing dramatically with more compounds available for more targets. And still, even a reduction of the above possible combinations by 99% will leave us with over 21,000 possible drug combinations.

The limitations evaluating combination therapy-based personalized medicine approaches described above lie in the conventional clinical trial design, which mainly relies on statistical comparisons between study groups. To facilitate statistical analyses in classical clinical trials, patient characteristics and therapy regimens have to be in narrow confines[28, 29]. However, the requirement of classical clinical trials that patients should be “similar” and groups “homogeneous”, which we will refer to as “homogeneity criterion”, is irreconcilable with the molecular diversity and diverse therapeutic options of the future personalized medicine approaches outlined above. The necessity to find sufficiently large numbers of similar patients is in stark contrast to the constantly growing number of molecular features effectively rendering cancer patients increasingly dissimilar.

In our opinion, the solution to the dilemma that personalized medicine will have to embrace complexity to thrive and at the same time cope with the limited suitability of conventional clinical trial designs to test complex therapies lies in a combination of improved tissue-based, functionality-oriented molecular characterization of tumors and novel design concepts for clinical trials. While the aim to understand mechanisms of disease has mainly been the domain of basic biomedical research, it will become increasingly necessary to follow a similar path in clinical research. Only improved knowledge of the biological implications of pathological alterations highjacking cellular processes will help reduce the level of (combinatorial) complexity and will allow for the rational pre-selection of a limited number of drug combinations instead of a systematic evaluation of all possible combinations or a mere random therapy selection which – as we have shown above – is like finding a needle in the hay stack.

**Figure 2:**
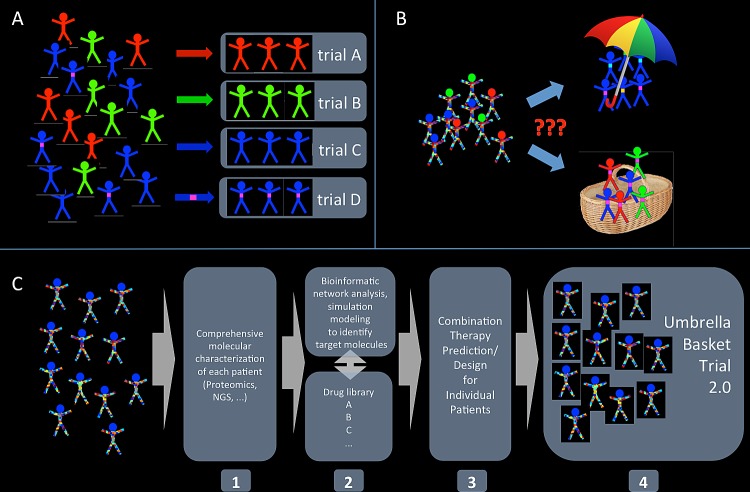
A novel approach to clinical trials: combining and extending basket and umbrella trials The classical approach recruiting patients according to a high-level diagnosis (e. g. lung adenocarcinoma “blue”) potentially refined by single markers (e. g. EML4-ALK-positive lung adenocarcinoma, “blue with magenta spot”) for statistical comparison between therapy groups (A) will fail with even a handful of druggable mutations under investigation (B) With an increasing number of actionable mutations and the need for combination therapies, even novel approaches such as basket or umbrella trials are incapable of addressing the arising combinatorial complexity. We therefore propose a concept draft for the development of a novel clinical trial design approach (C) that incorporates 1) a comprehensive functional analysis of the molecular tumor features that are 2) subsequently analyzed using bioinformatics and computational modeling of the (pathologically altered) network to identify target molecules. In combination with a drug library this knowledge is 3) used to propose optimal combination therapies for each patient who is then 4) recruited to the trial in which multiple different combination therapies are assessed. Such trials test the efficacy of the molecular analysis and therapy selection method and the employed drug library and therefore provide an implicit therapy validation.

However, even a leap forward in the mechanistic understanding of cancer biology fostering a more rational therapy design will certainly not reduce the relevance of clinical trials. But to address the complexity issues, clinical trials will have to extend beyond the “homogeneity criterion”-based design as described above. While “basket” and “umbrella” approaches are a step in the right direction, because they move beyond the boundaries of single organs and therapies, our combinatorial considerations discussed above show that they can only be an interim solution, as they do not solve the complexity problem. Moreover, limiting molecular diagnostics to mutational profiling may neglect functional aspects and lead to an ineffective therapy[30]. As a potential solution we suggest to combine and extend design features of basket and umbrella trials by performing a comprehensive functional molecular characterization of the tumor followed by systems biology driven analysis to identify drug targets and propose efficacious combination therapies. Such systems analysis will integrate genomics and proteomics methods with advanced bioinformatics and simulation modeling using information on the underlying network structure. Another important aspect of this approach will be the availability of compound libraries (provided by pharmaceutical companies) that contain drugs approved for use in humans to target the different (pathologically altered) signaling network components. Within the limits of available actionable mutations and corresponding targeted drugs, the novel clinical trials need not comply with the homogeneity criterion and may recruit patients even with diverse molecular phenotypes. The concept of clinical molecular analysis that we propose will help identify targets and propose custom-tailored targeted therapies for individual patients. While reports on the success or failure of a particular individual therapy accompanied by a molecular rationale for the clinical observation will advance our understanding of precision medicine and may help deduce mechanisms underlying treatment response, they remain anecdotal[31-34] and eventually provide insufficient evidence for the general efficacy of a specific therapy. In contrast, evaluating multiple different individual therapies designed by a reproducible approach may offer a solution. The combined “umbrella-basket” trial design will facilitate the evaluation of different therapies for different cancers. Such clinical trials would not primarily evaluate the efficacy of a particular combination therapy, but would verify the systems biology approach in combination with the respective drug library. In other words, this new type of clinical trial would verify the method with which targeted (combination) therapies are selected for individual patients for a set of actionable mutations and a corresponding library of targeted drugs. This “indirect” validation relies on mechanistic rationales instead of combinatorial designs and moreover, allows for a significant reduction in the number of patients needed to be screened (Figure 2).

A current limitation of the proposed approach is that it strongly relies on detailed knowledge of disease mechanisms that will have to be achieved through technically difficult strategies. Other disadvantages compared to classical trials might be the heterogeneous efficacy of the drugs or unknown interaction effects specific to a particular drug combination. On the other hand, the proposed novel approach would be designed to provide improved therapies for individual patients as opposed to the averaged effects determined in classical clinical trials. “What is beneficial for the average may not help the individual patient” even in case of a successful clinical trial will turn into “the approach for finding the right therapy for an individual patient can also be used to find personalized therapies beneficial for other patients”, whereas “beneficial” in the first case refers to a single pre-defined therapy, but to the method with which the right therapy is found in the second case.

To conclude, in this discussion we do not aim at presenting a ready-to-use solution, which will require significant efforts over the next few years in the field of companion diagnostics and systems medicine research, pharmaceutical drug development and targeted drug testing as well as concerted efforts involving regulators, scientists and physicians to integrate these diverse aspects. However, with our quantitative analysis of the emerging combinatorial complexity of targeted therapies described herein we hope to have presented arguments and a roadmap for a fundamentally different clinical trial design to better fulfill the promise of personalized medicine in cancer medicine.
